# Supplementary material for: Evolutionary and biochemical analyses reveal conservation of the Brassicaceae telomerase ribonucleoprotein complex
Source: PLoS One. 2020 Apr 9;15(4):e0222687. doi: 10.1371/journal.pone.0222687 (PMC7145096; doi:10.1371/journal.pone.0222687)
Supplement: S1 Fig — Roman numerals correspond to branches tested in Fig 2. Alternative model likelihood scores that are significantly greater than null model likelihood scores indicate positive selection (p<0.05) along the specified branch and are denoted in bold. (PDF) [file pone.0222687.s001.pdf]

Supplemental Figure 1

Gene	Branch	Null (ω ≤ 1)	Alt (1 ≤ ω)	Alt-Null	Liklihood ratio score (2[Alt-Null])	Significance
Dyskerin	i	-6102.87	-6102.87	0	0	$p > 0.05$
	ii	-6102.85	-6102.85	0	0	$p > 0.05$
	iii	-6100.38	-6096.32	4.06	**8.13**	**$p \leq 0.01$**
	iv	ND	ND	ND	ND	ND
	v	ND	ND	ND	ND	ND
	vi	-6096.37	-6094.80	1.57	**3.14**	**$p \leq 0.05$**
Ku70	i	-5799.49	-5799.49	0.00	0.00	$p > 0.05$
	ii	-5798.94	-5796.68	2.26	**4.52**	**$p \leq 0.05$**
	iii	-5799.49	-5799.49	0.00	0.00	$p > 0.05$
	iv	ND	ND	ND	ND	ND
	v	ND	ND	ND	ND	ND
	vi	-5799.49	-5799.49	0.00	0.00	$p > 0.05$
Ku80	i	-7725.67	-7725.67	0.00	0.00	$p > 0.05$
	ii	-7725.67	-7725.67	0.00	0.00	$p > 0.05$
	iii	-7725.67	-7725.67	0.00	0.00	$p > 0.05$
	iv	-7724.92	-7724.92	0.00	0.00	$p > 0.05$
	v	ND	ND	ND	ND	ND
	vi	-7725.67	-7725.67	0.00	0.00	$p > 0.05$
