# Supplementary material for: Evolutionary and biochemical analyses reveal conservation of the Brassicaceae telomerase ribonucleoprotein complex
Source: PLoS One. 2020 Apr 9;15(4):e0222687. doi: 10.1371/journal.pone.0222687 (PMC7145096; doi:10.1371/journal.pone.0222687)

Supplemental Figure 2

A

	Expected Product Difference (EPD) Relative to N15-GGG		Observed (for *A. thaliana*)
1:	N15-NNNNAG**GG**<br>3'-AUCCCAAAUC-5'	**= +5**	**+4**
2:	N15-NNNNGG**G**<br>3'-AUCCCAAAUC-5'	**= +4**	**+4**
3:	N15-NNNGGG<br>3'-AUCCCAAAUC-5'	**= +3**	**+3**
4:	N15-GGG<br>3'-AUCCCAAAUC-5'	**= +0**	**+0**
5:	N15-NNNNTT**TAGGG**<br>3'-AUCCCAAAUC-5'	**= +1**	**+1**
6:	N15-NNNNNT**TTAGGG**<br>3'-AUCCCAAAUC-5'	**= +2**	**+6**
7:	N15-NNT**TTAGGG**<br>3'-AUCCCAAAUC-5'	**= +6**	**+3**

B

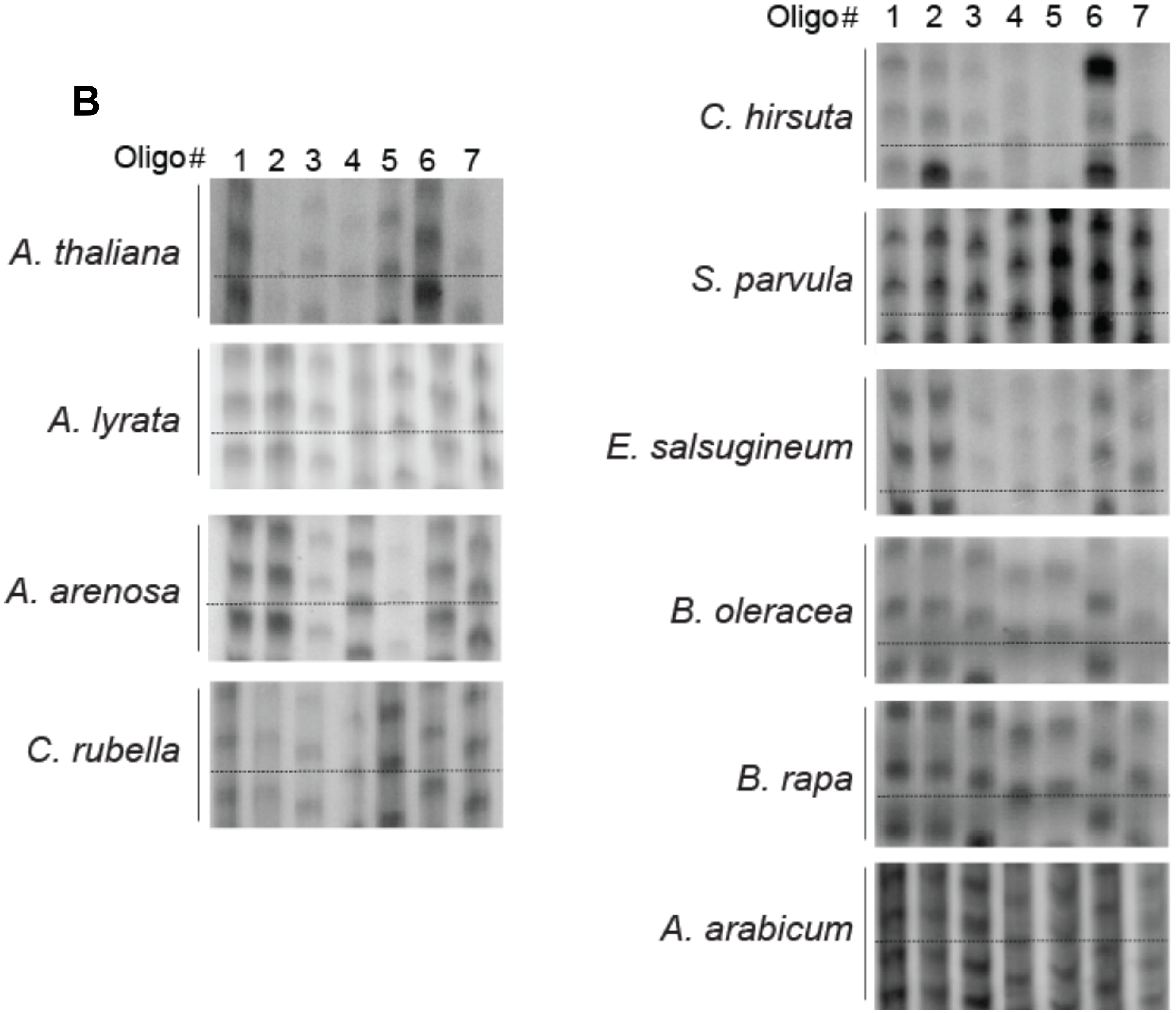

Supplement: S2 Fig — (A). Expected binding between AtTER1 template and each oligo used in the experiment. The first set of nucleotides added by this template before the first full repeat is shown in bold. Expected product differences are calculated relative to oligo #4, which is the shortest oligo provided in the assay and therefore serves as the baseline. (B) A subset of the gel image from the substrate utilization assay for each species tested. A dashed line is drawn through the center of a band for oligo N15-GGG, which serves as the baseline for calculating observed product differenced for the other oligos. (PDF) [file pone.0222687.s002.pdf]
