# Supplementary figures and images for: Evolutionary and biochemical analyses reveal conservation of the Brassicaceae telomerase ribonucleoprotein complex
Source: PLoS One. 2020 Apr 9;15(4):e0222687. doi: 10.1371/journal.pone.0222687 (PMC7145096; doi:10.1371/journal.pone.0222687)

Supplemental Figure 3

A

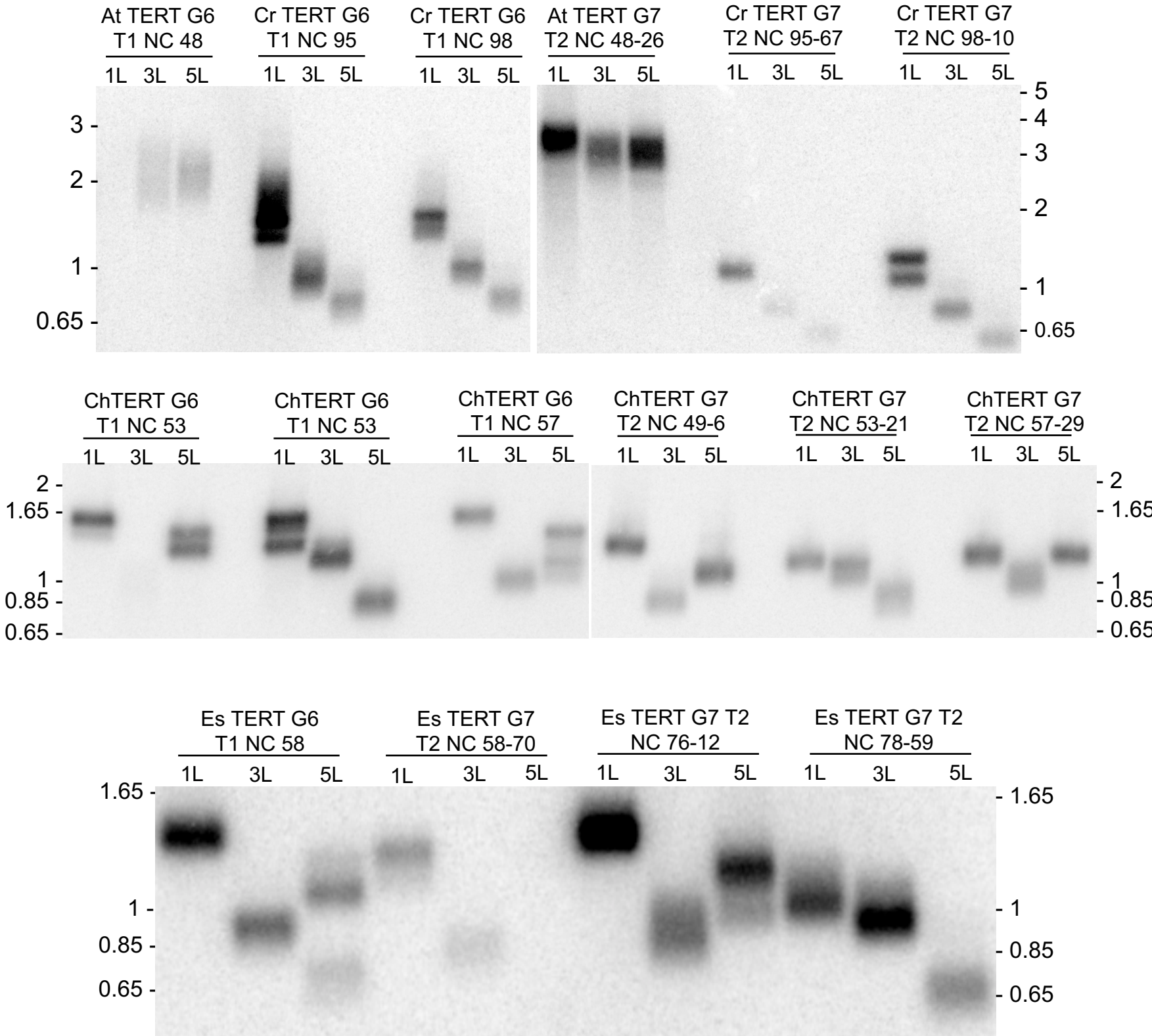

Supplemental Figure 3

B

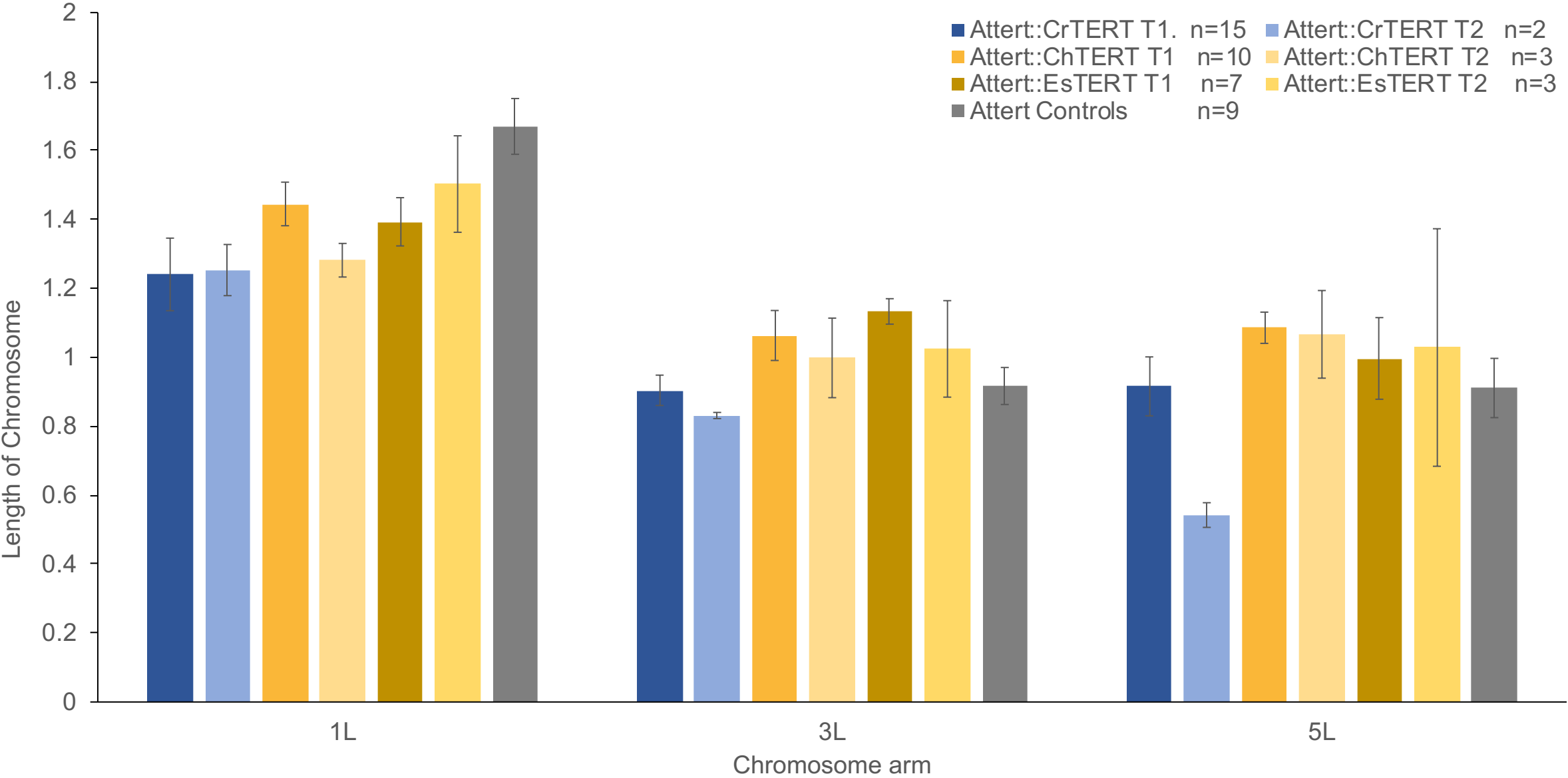

Supplemental Figure 3

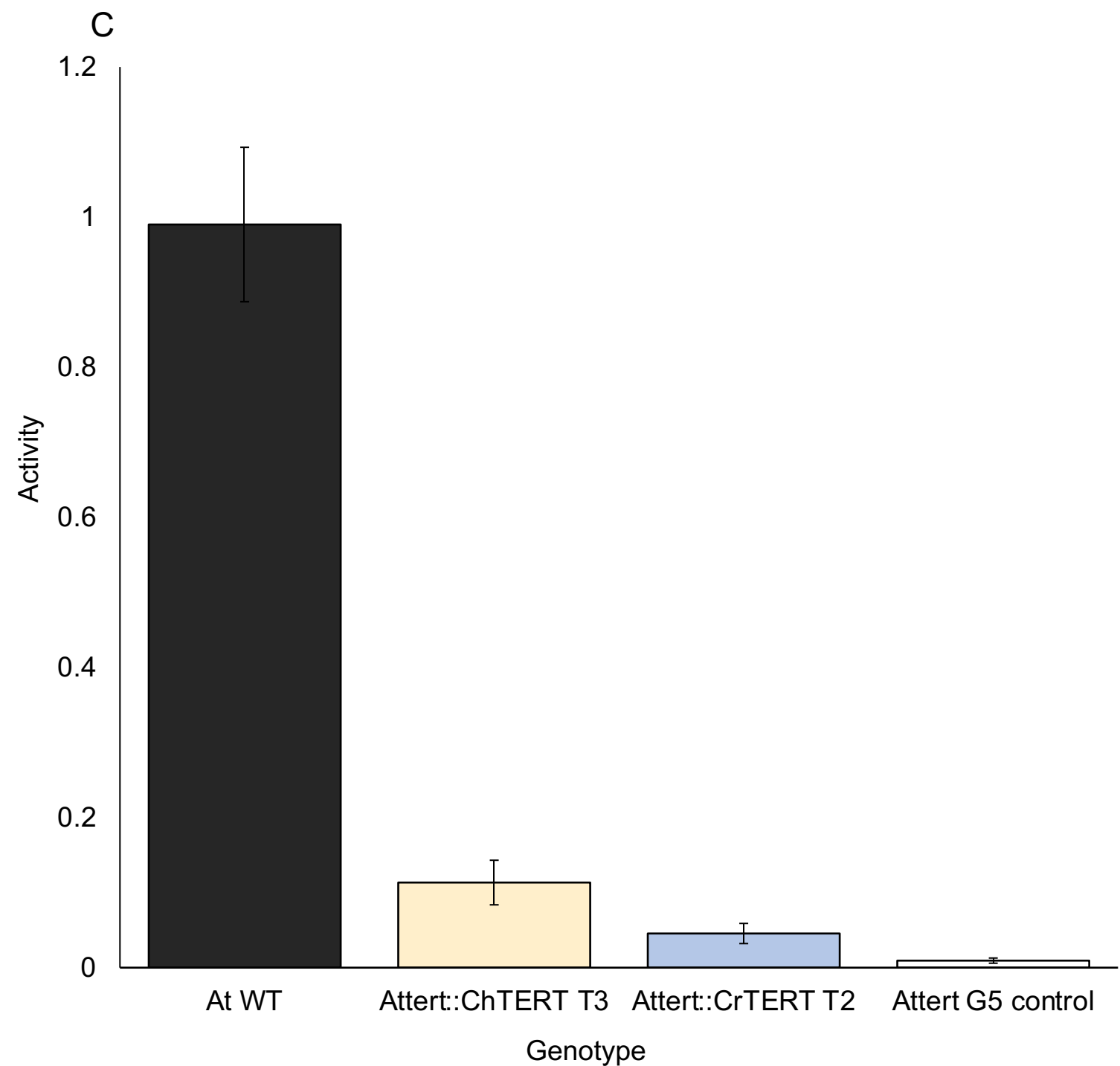

Supplement: S3 Fig — (A) PETRA results for Attert -/- lines transformed with C. rubella TERT (Cr), C. hirsuta TERT (Ch), and E. salsugineum TERT (Es). (B) Quantification of results shown in (A) and an unselected Attert -/- control. Error bars represent standard error. (C) Telomerase activity relative to wild type. All samples were significantly different (p < 0.001) from the Attert G5 control using a Student’s t-test with a Benjamini-Hochberg multiple testing correction with error bars representing standard deviation. For all experiments, n ≥ 3. (PDF) [file pone.0222687.s003.pdf]

Supplemental Figure 4

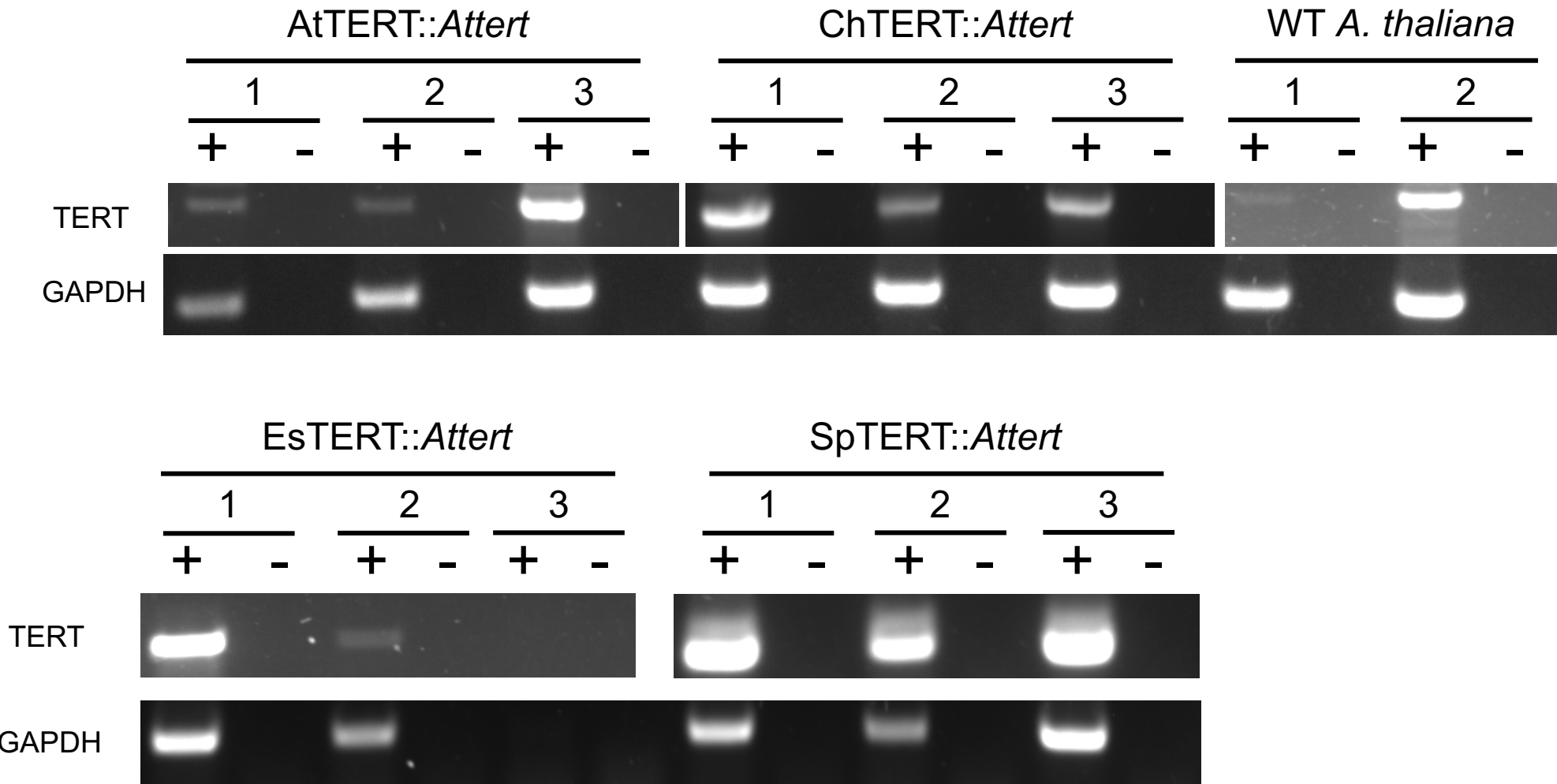

Supplement: S4 Fig — RNA was extracted from floral tissue from each background. RT-PCR was performed on both the 3’ end of the TERT transgene (shown) and the full-length construct (not shown). GAPDH was used to determine quality of RNA and as an approximate loading control. “+” indicates a positive RT reaction, whereas “-”indicates no reverse transcriptase was added. (PDF) [file pone.0222687.s004.pdf]
