# Supplementary material for: Evolutionary and biochemical analyses reveal conservation of the Brassicaceae telomerase ribonucleoprotein complex
Source: PLoS One. 2020 Apr 9;15(4):e0222687. doi: 10.1371/journal.pone.0222687 (PMC7145096; doi:10.1371/journal.pone.0222687)

Figure 1B

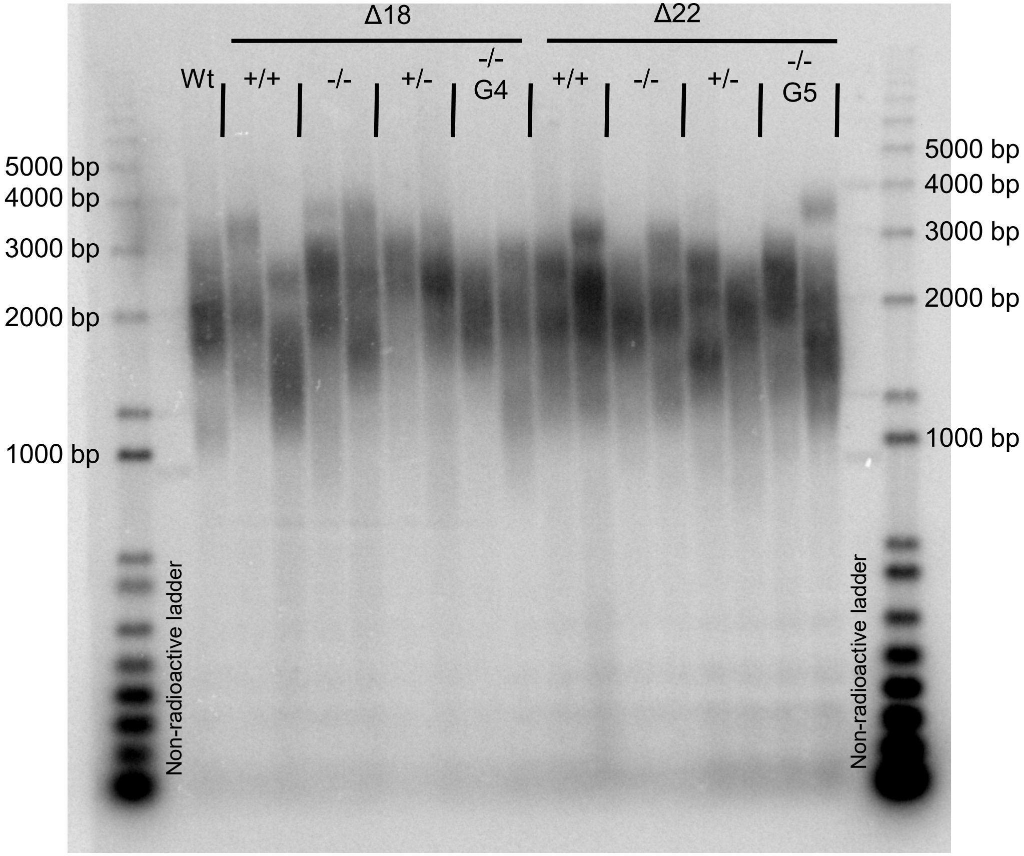
Δ18
Δ22
Wt
+/+
-/-
+/-
-/-
G4
+/+
-/-
+/-
-/-
G5
5000 bp
4000 bp
3000 bp
2000 bp
1000 bp
5000 bp
4000 bp
3000 bp
2000 bp
1000 bp
Non-radioactive ladder
Non-radioactive ladder

Figure 1C

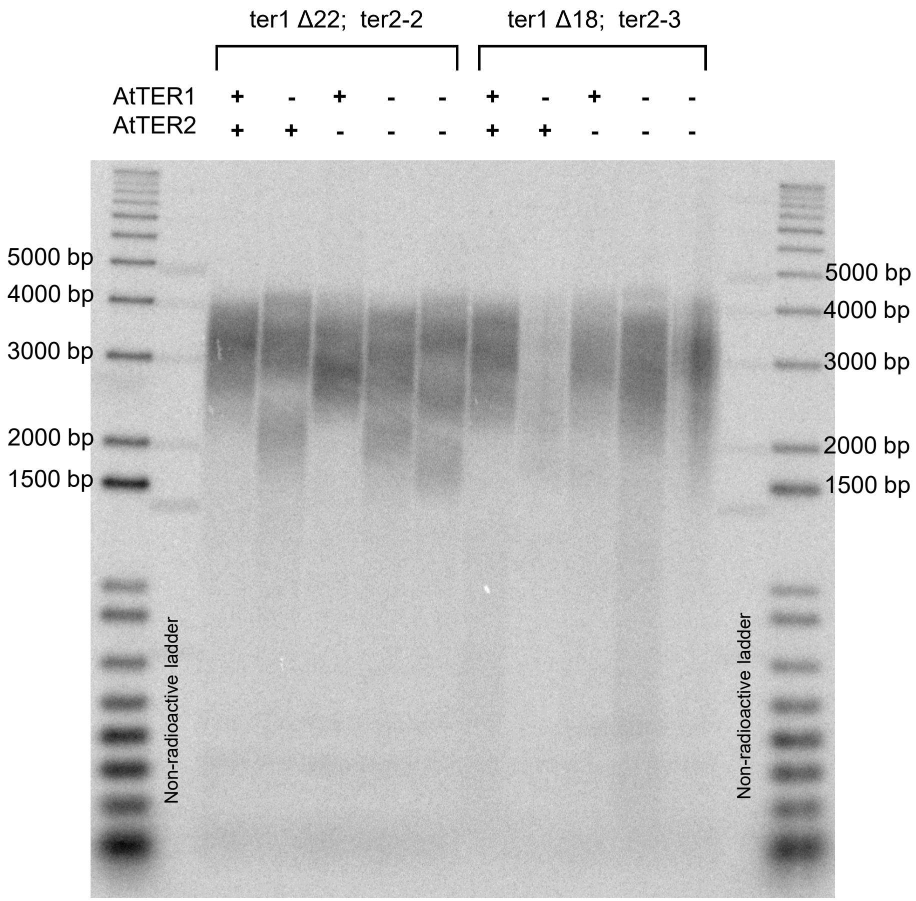
ter1 Δ22; ter2-2
ter1 Δ18; ter2-3
AtTER1 + - + - - + - + - -
AtTER2 + + - - - + + - - -
5000 bp
4000 bp
3000 bp
2000 bp
1500 bp
Non-radioactive ladder
Non-radioactive ladder
5000 bp
4000 bp
3000 bp
2000 bp
1500 bp

Figure 4A

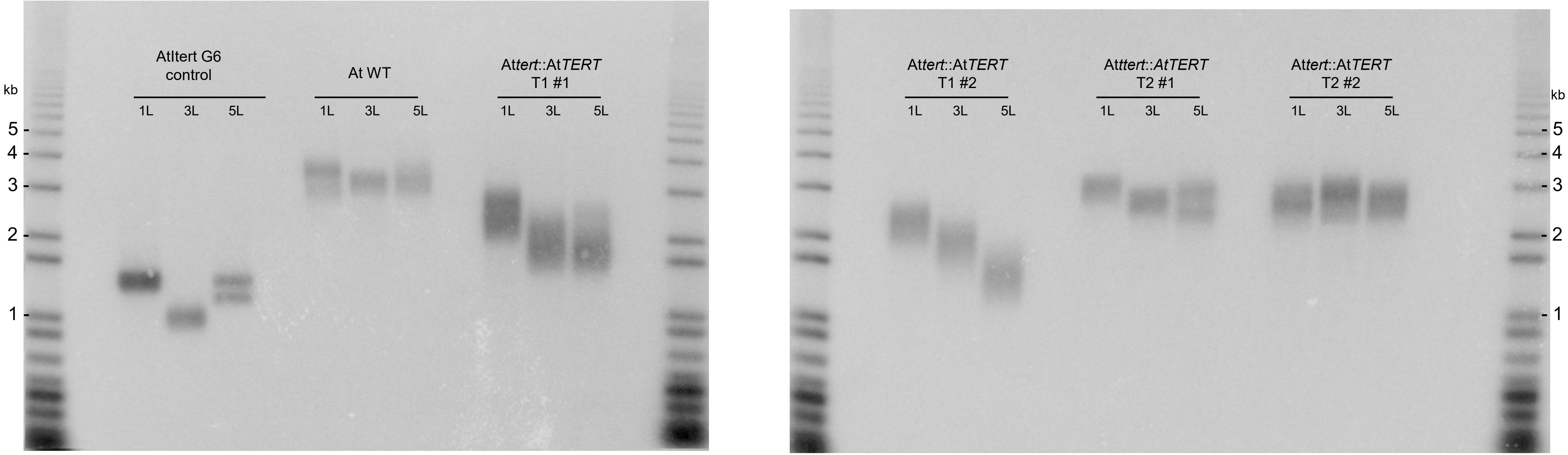

AtItert G6 control
At WT
Attert::AtTERT T1 #1
Attert::AtTERT T1 #2
Attert::AtTERT T2 #1
Attert::AtTERT T2 #2
1L 3L 5L
kb
5
4
3
2
1

Figure 4B

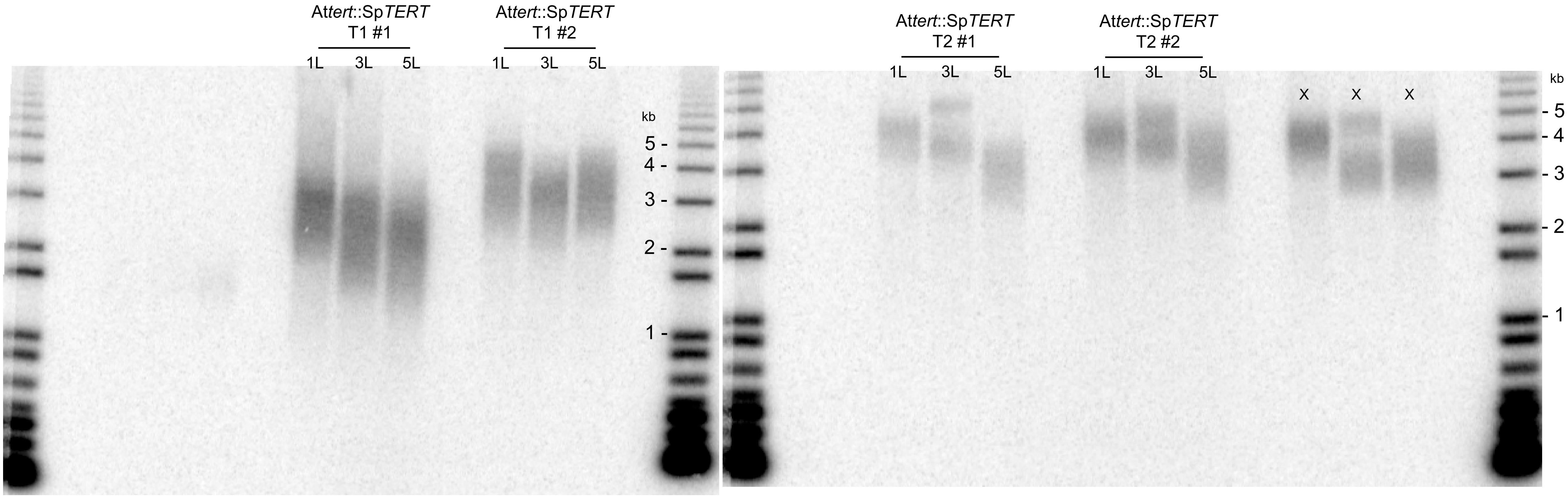
Attert::SpTERT
T1 #1
1L 3L 5L
Attert::SpTERT
T1 #2
1L 3L 5L
kb
5 -
4 -
3 -
2 -
1 -
Attert::SpTERT
T2 #1
1L 3L 5L
Attert::SpTERT
T2 #2
1L 3L 5L
X X X
kb
- 5
- 4
- 3
- 2
- 1

Figure 5C

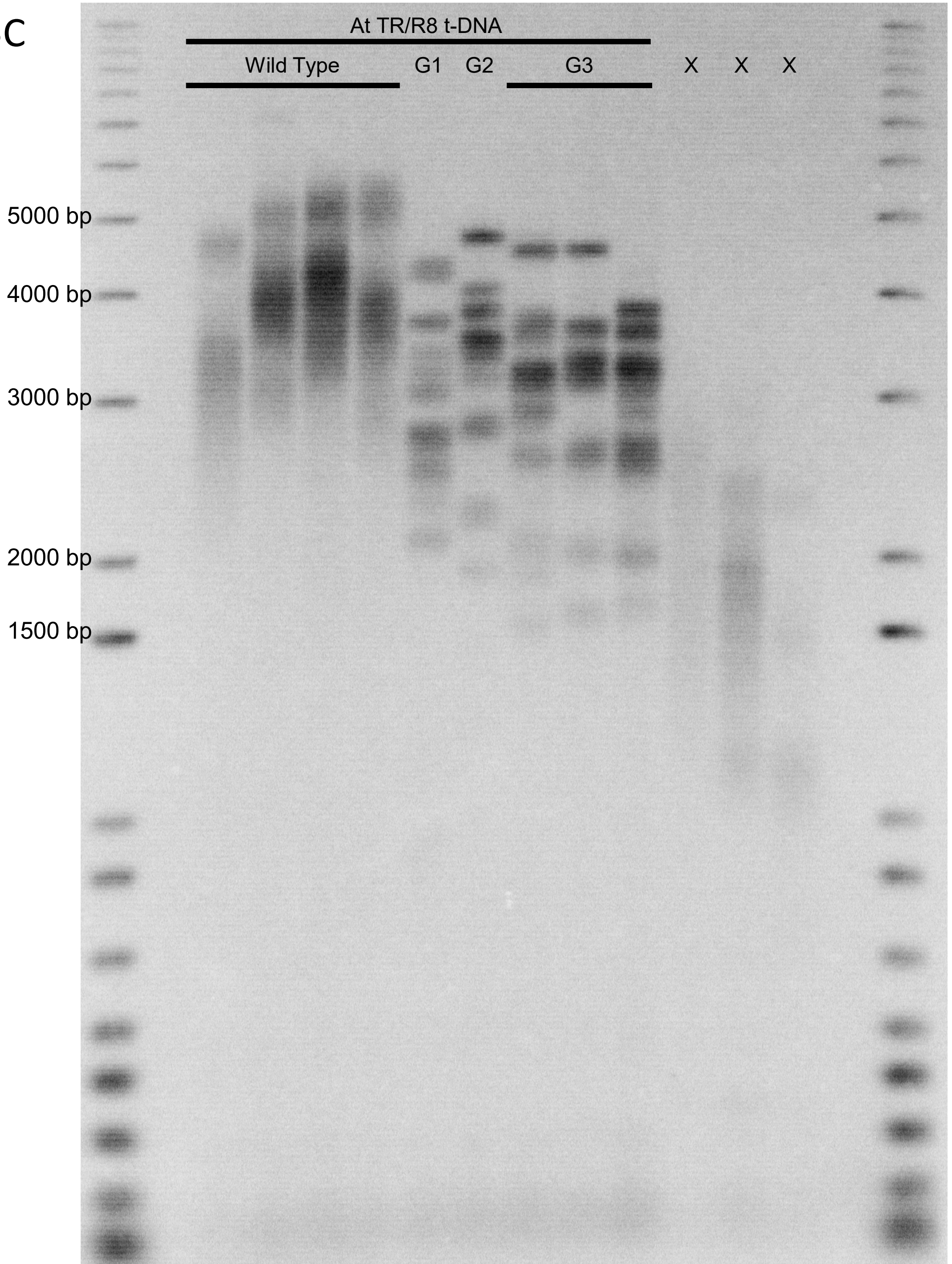

At TR/R8 t-DNA
Wild Type
G1
G2
G3
X
X
X
5000 bp
4000 bp
3000 bp
2000 bp
1500 bp

Supplemental Figure 2B

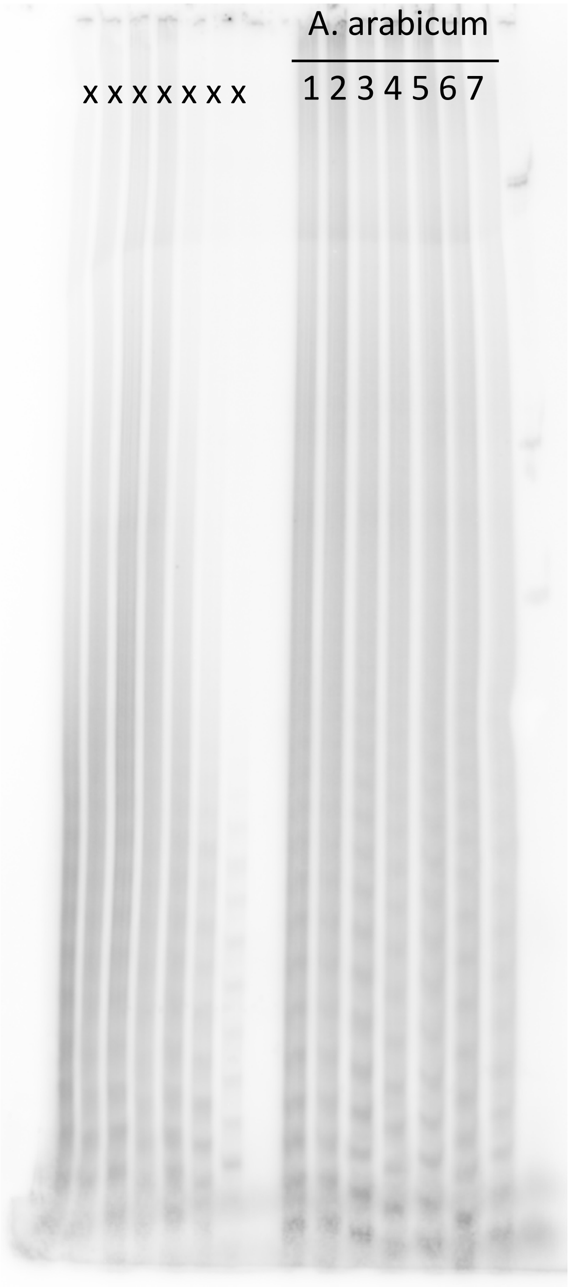

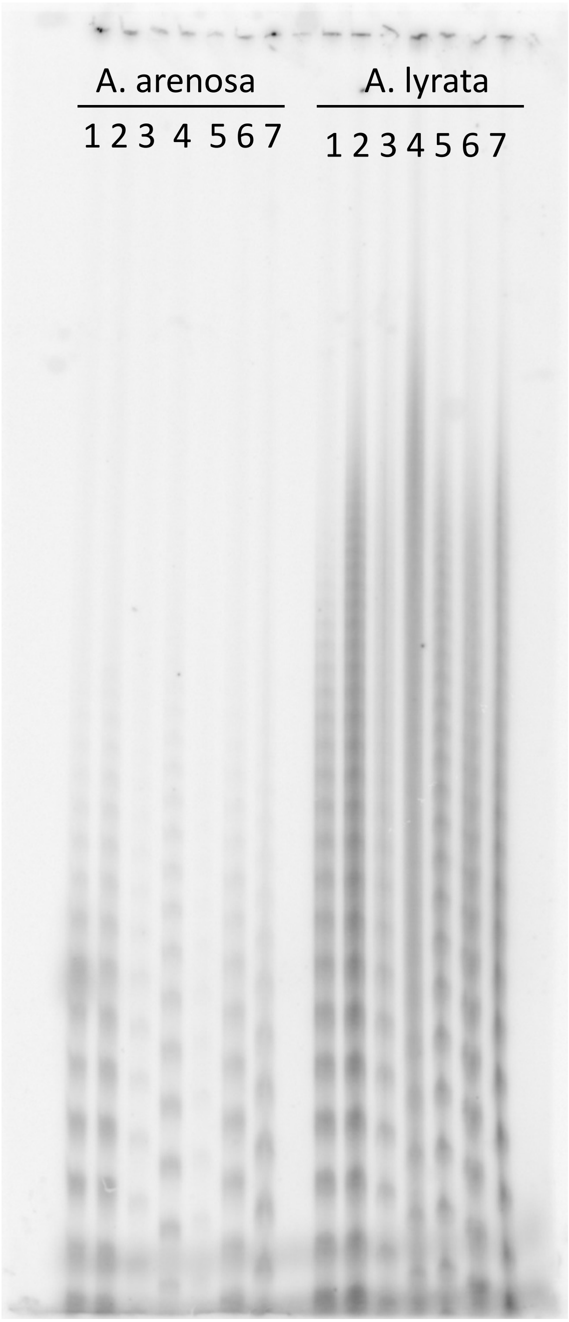

Supplemental Figure 2B

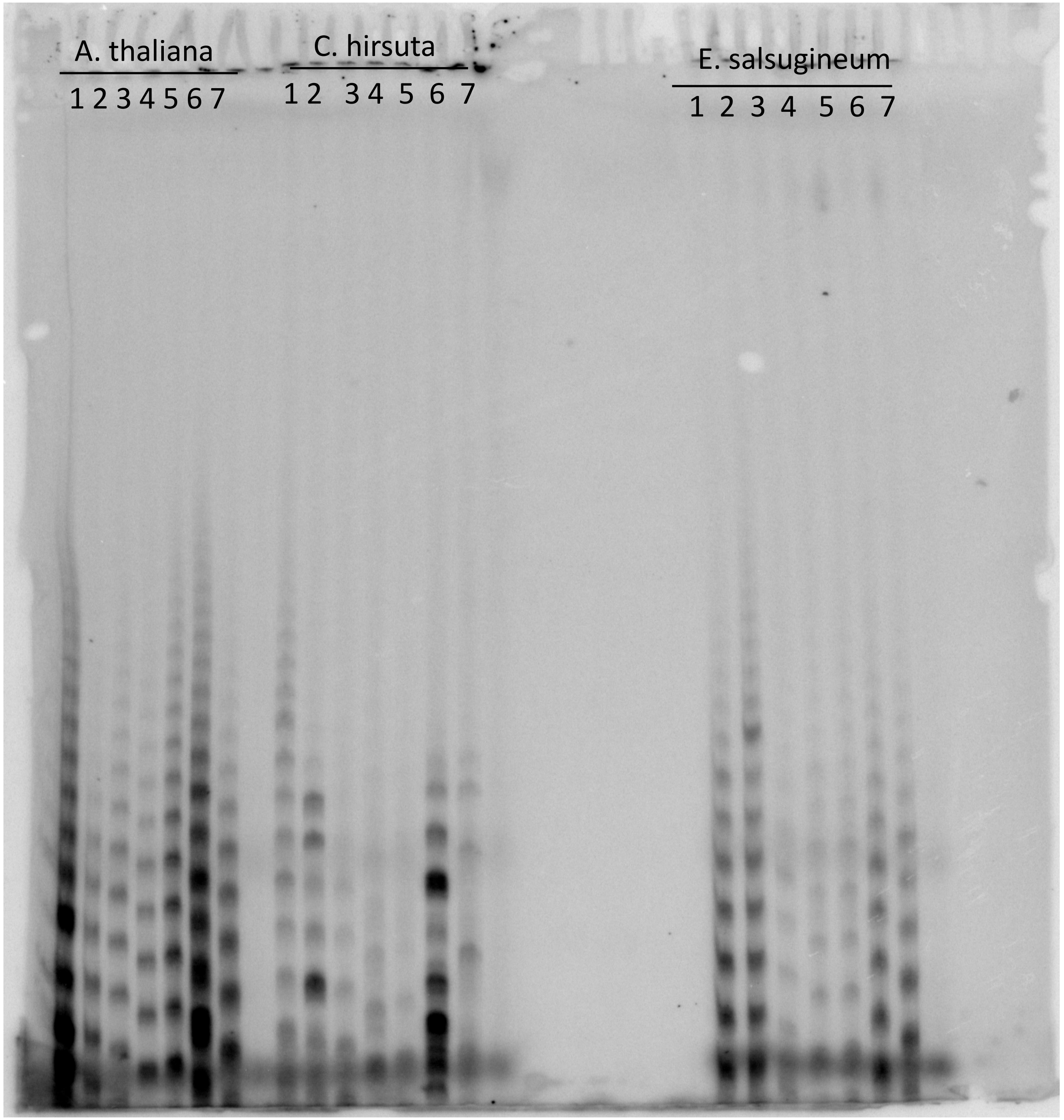

Supplemental Figure 2B

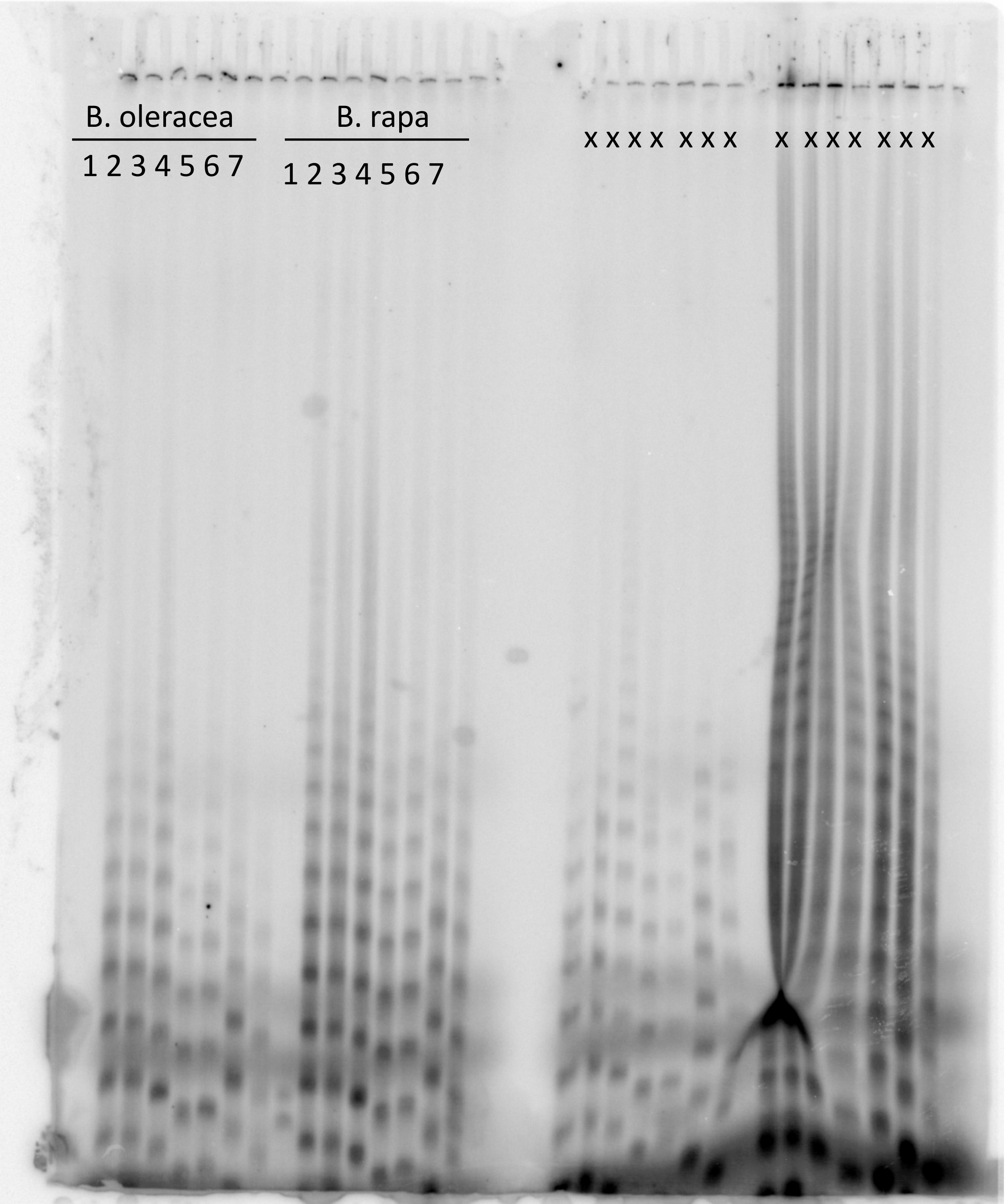

Supplemental Figure 2B

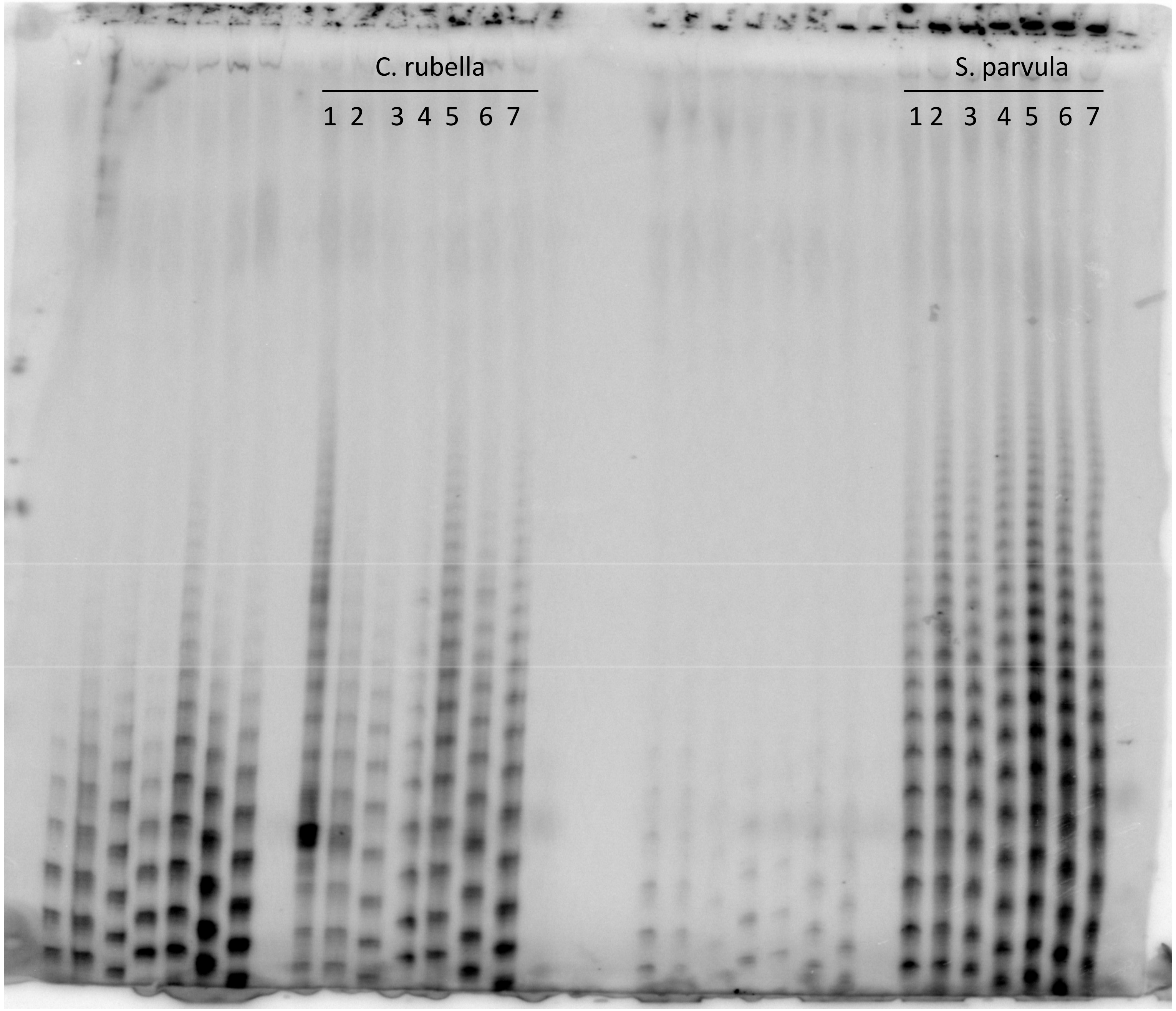

Supplemental Figure 3A

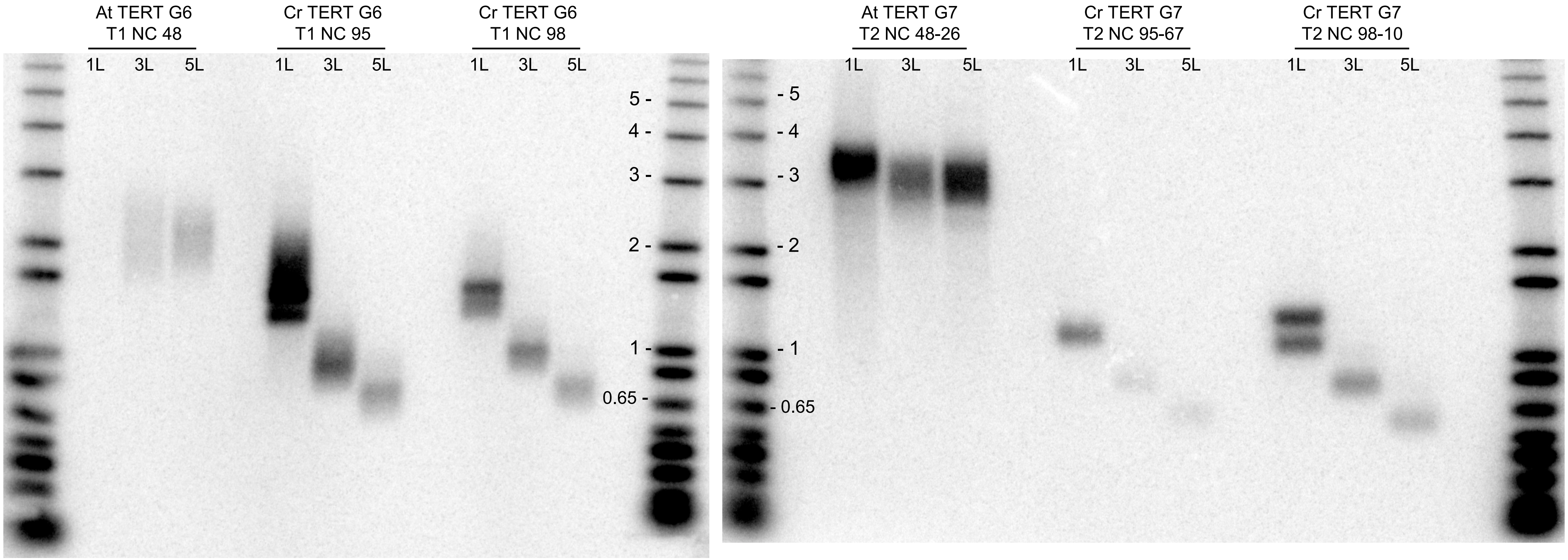

Supplemental Figure 3A

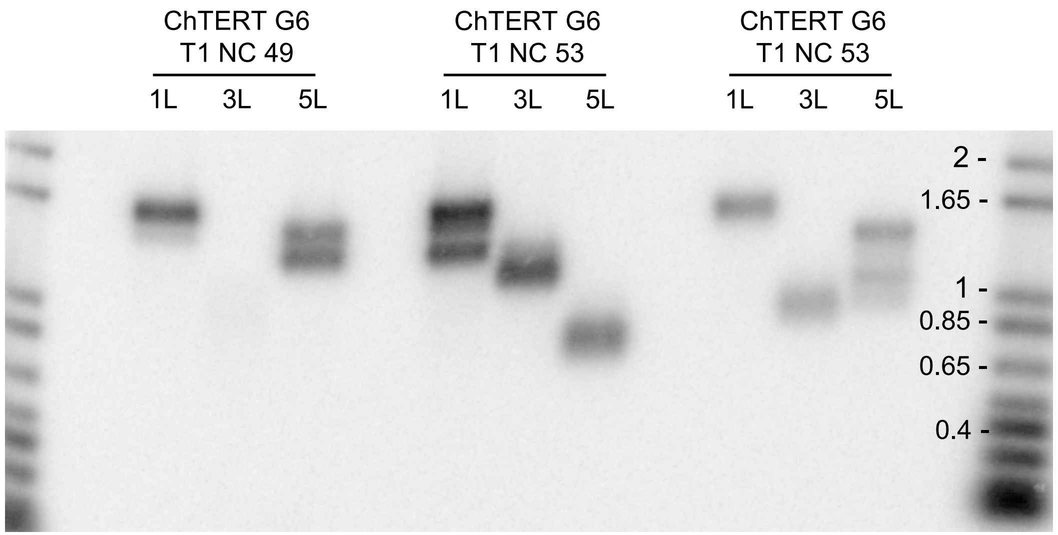

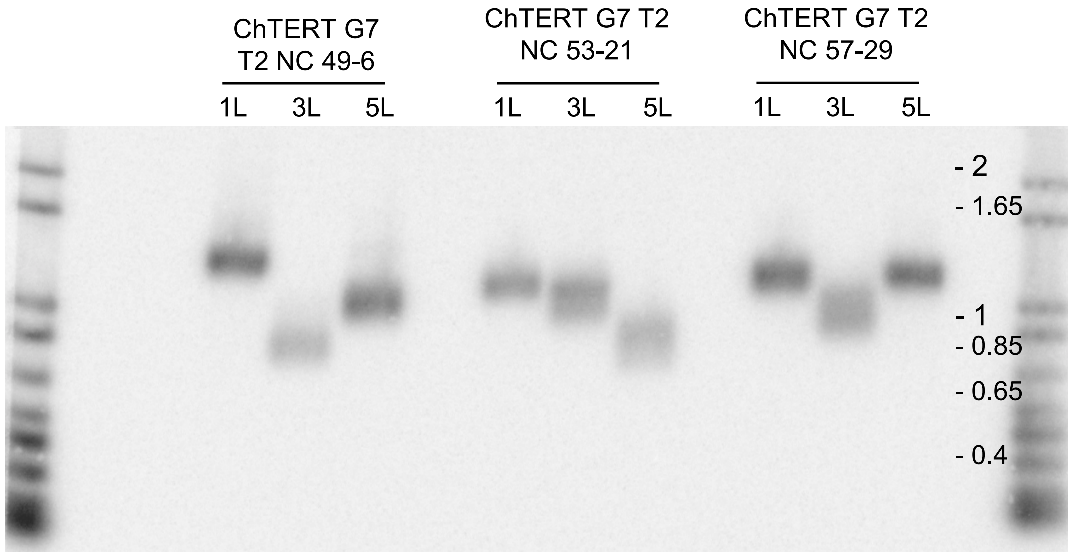

Supplemental Figure 3A

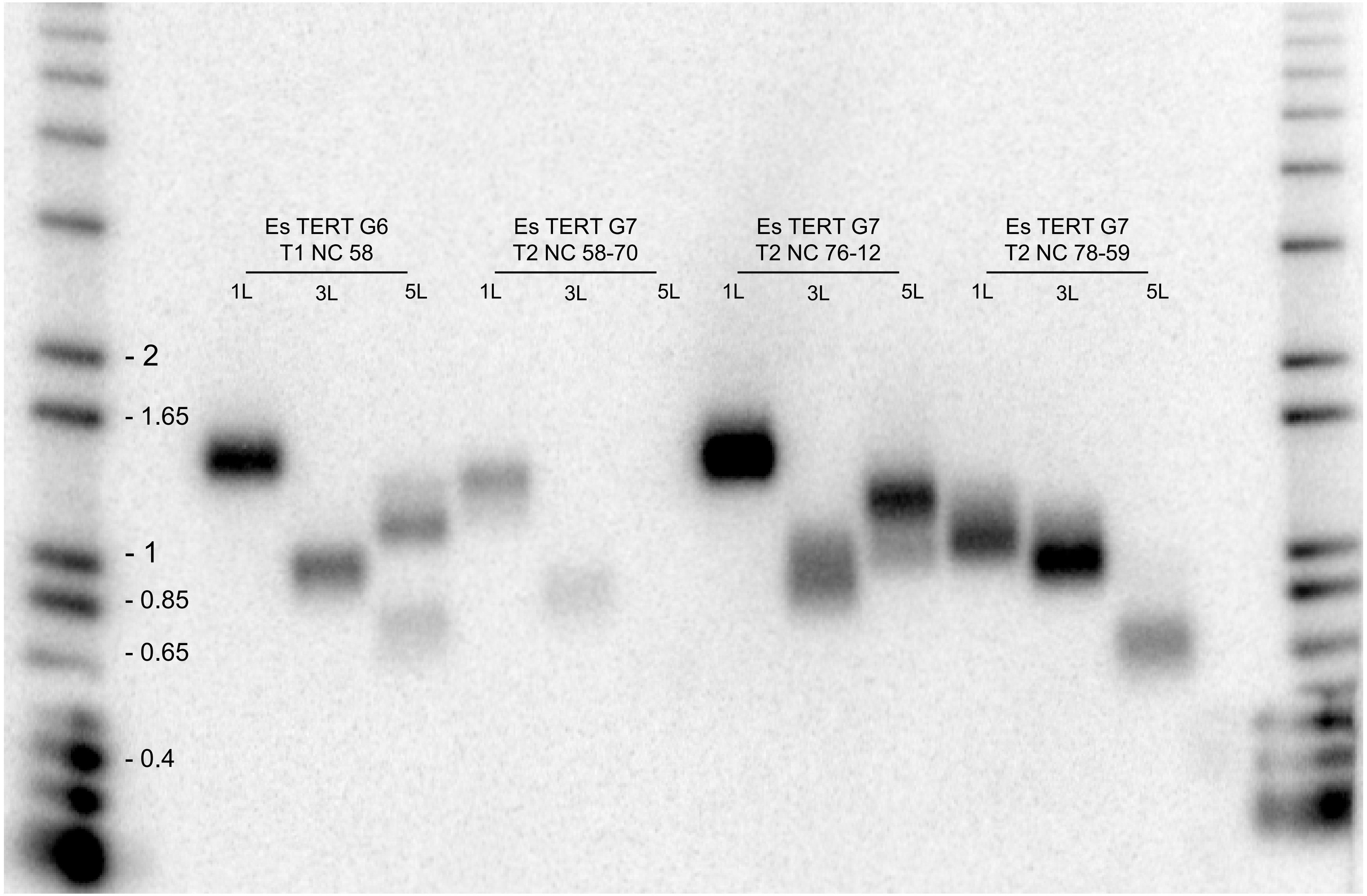

Supplemental Figure 4 TERT

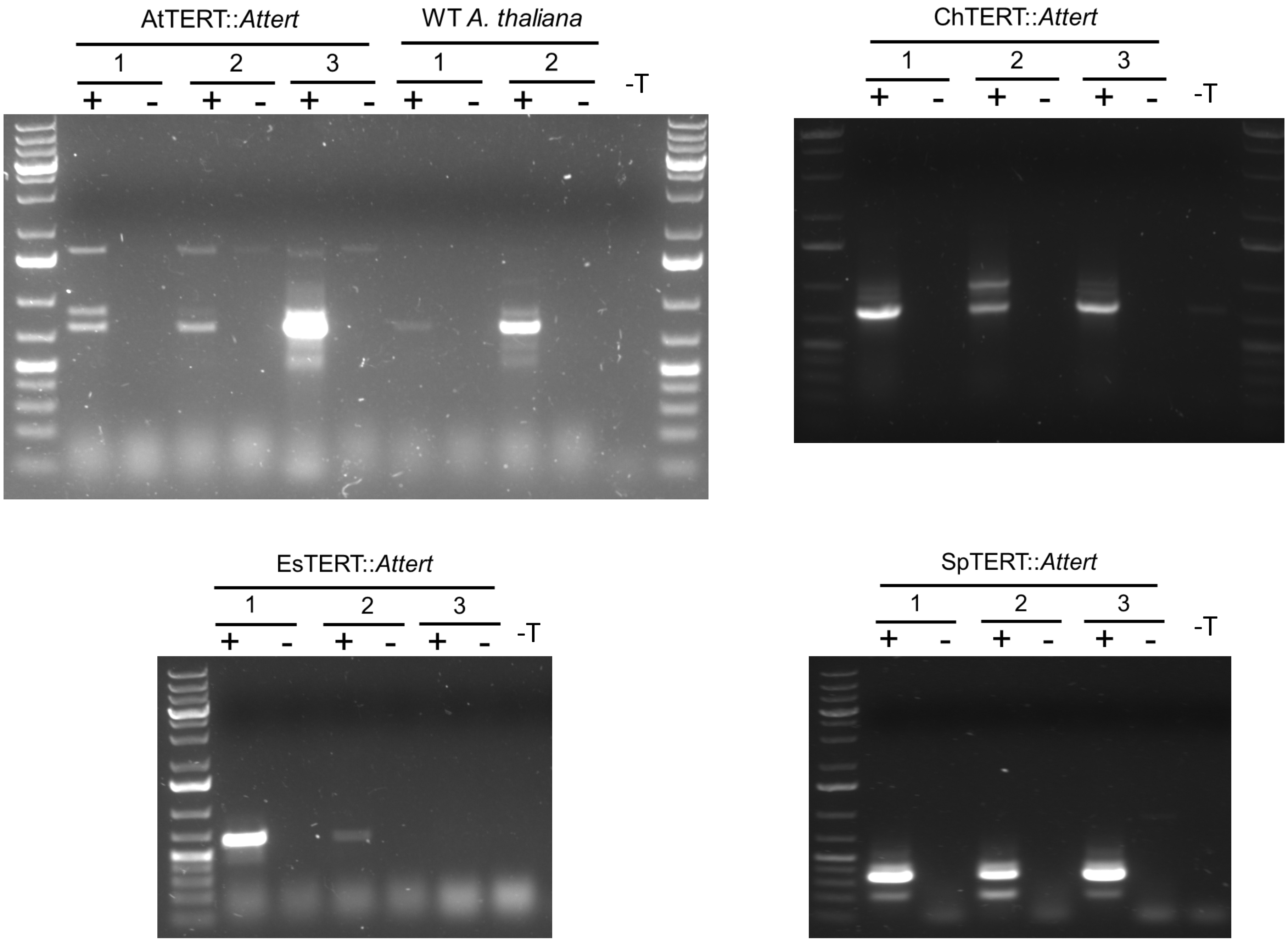

Supplemental Figure 4 GAPDH

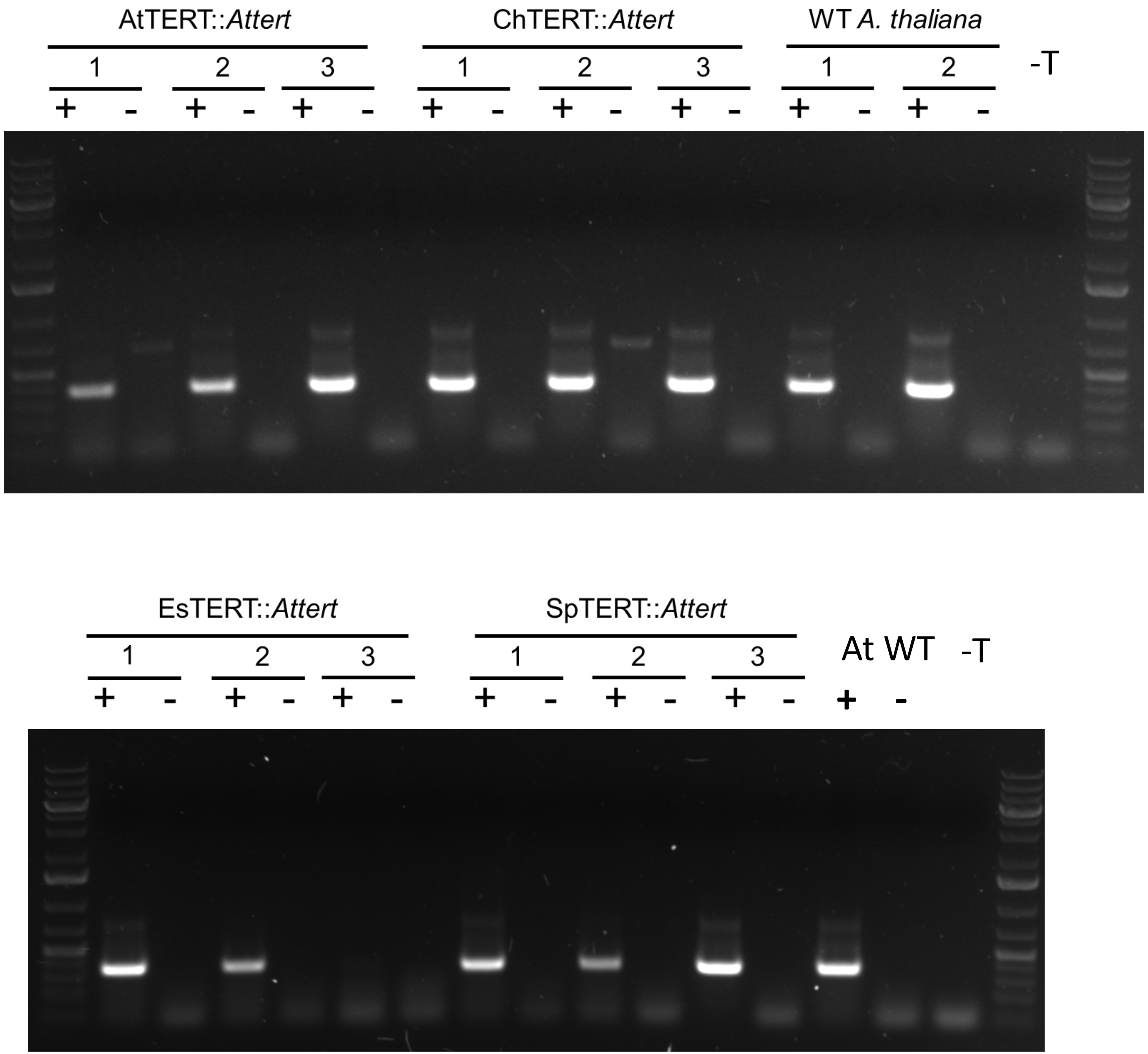

Supplement: S1 Raw Images — (PDF) [file pone.0222687.s006.pdf]
